# Selective activity of extracts of *Margaritaria discoidea *and *Homalium africanum *on *Onchocerca ochengi*

**DOI:** 10.1186/1472-6882-10-62

**Published:** 2010-10-28

**Authors:** Fidelis Cho-Ngwa, Melanie Abongwa, Moses N Ngemenya, Kennedy D Nyongbela

**Affiliations:** 1 Department of Biochemistry and Microbiology, and Department of Chemistry, Faculty of Science, University of Buea, P.O. Box 63, Buea, Cameroon

## Abstract

**Background:**

The current treatment of onchocerciasis relies on the use of ivermectin which is only microfilaricidal and for which resistant parasite strains of veterinary importance are increasingly being detected. In the search for novel filaricides and alternative medicines, we investigated the selective activity of crude extracts of *Margaritaria discoidea *and *Homalium africanum *on *Onchocerca ochengi*, a model parasite for *O. volvulus*. These plants are used to treat the disease in North West Cameroon.

**Methods:**

Sixteen crude extracts were prepared from various parts of *M. discoidea *and *H. africanum *using different organic solvents. The filaricidal activities were determined *in vitro*. Cytotoxicity of the active extracts was assessed on monkey kidney epithelial cells *in vitro *and the selectivity indices (SI) of the extracts determined. Acute toxicity of the promising extracts was investigated in mice.

**Results:**

Four out of the 16 extracts showed microfilaricidal activity based on motility reduction, whereas, none showed macrofilaricidal activity based on the MTT/formazan assay. The methylene chloride extract of *H. africanum *leaves (HLC) recorded the lowest IC_50 _of 31.25 μg/mL and an IC_100 _of 62.5 μg/mL. The SI for the active extracts ranged from 0.5 - 2.63. No form of acute toxicity was observed in mice. Phytochemical analysis revealed the presence of anthraquinones, sterols and terpenoids in the promising extracts.

**Conclusions:**

The non-polar extracts of *M. discoidea *and *H. africanum *are potential sources of new microfilaricidal lead compounds, and the results support their use in traditional medicine.

## Background

Onchocerciasis is a parasitic disease of man caused by the filarial worm, *Onchocerca volvulus*, and is transmitted by the black fly, *Simulium damnosum *[1]. It remains a major public health problem with over 37 million patients and a risk population of over 120 million [2]. Chemotherapy remains the main method of control of onchocerciasis. Ivermectin being the only drug currently recommended for treatment of the disease effectively kills the microfilariae of *O. volvulus*, but not the adult worms (macrofilariae) which may live for up to 18 years in humans [3]. Also, the emergence of ivermectin resistance in parasitic nematodes of veterinary importance [4] raises serious concerns that this may extend to the human *O. volvulus*. Therefore, the search for new and highly efficacious filaricides is imperative.

Medicinal plant preparations have been identified as alternative remedies for several diseases [5]. About 50% of drugs used in modern medicine are of plant origin [6] and about 80% of Africa's population rely on medicinal plants for their health needs [7]. Filaricidal activities have been detected in some plants [8,9]. However, because of the toxicity of extracts of some of these plants, obtaining initial cytotoxicity data should be important in guiding the preparation of phytomedicines and in making a decision on the specificity of the anti-parasitic activity. *Margaritaria discoidea *and *Homalium africanum *were chosen for this study based on their acclaimed effectiveness in the treatment of onchocerciasis by traditional medicine practitioners in North West Cameroon. In the area, the dried plant material is ground into powder which is consumed directly, or boiled in water and the decoction drunk. *Margaritaria discoidea *is used traditionally to treat several diseases in many countries, including certain helminth infections in Central Africa [10]. This study was therefore aimed at investigating the claimed filaricidal activities, and the toxicities of crude extracts of these plants which could serve as sources of new lead compounds for the development of much needed and efficacious drugs against onchocerciasis.

## Methods

### Collection and identification of plants

Plant parts from *M. discoidea *[Baill] Webster (Euphorbiaceae) and *H. africanum *[Hook. f] Benth (Flacourtiaceae) were collected from Bambili and Fingi villages in the North West Region of Cameroon in April 2008 based on ethnopharmacological information. The local names of these plants are "Sambarehi" and "Balerinyamnyam" for *M. discoidea *and *H. africanum *respectively. Voucher specimens were taken to the Limbe Botanic Garden (P.O Box 437 Limbe, Cameroon) where they were authenticated by a botanist, Mr. Litonga Ndive Elias, and assigned voucher numbers: S.C.A, Edwards *et al*. N° 5840 and S.C.A 438, N° 309 for *M. discoidea *and *H. africanum *respectively. Other relevant details on these plants are shown in Table 1.

**Table 1:** Plant parts and yield of crude extracts obtained with the different solvents

Plant part (dry weight in grams)		Extracting solvent			
		
		Hexane (H)	Methylene chloride (C)	Ethyl acetate (E)	Methanol (M)
***Margaritaria discoidea***					
**Leaves (L)** **(127.6)**	**Code**	MLH	MLC	MLE	MLM
	**% Yield**	0.86	1.57	0.94	2.12
**Stem bark (B)** **(441.7)**	**Code**	MBH	MBC	MBE	MBM
	**% Yield**	0.18	0.36	0.63	0.79
**Roots (R)** **(342.8)**	**Code**	MRH	MRC	MRE	MRM
	**% Yield**	0.58	0.26	0.20	0.20
***Homalium africanum***					
**Leaves (L)** **(164.0)**	**Code**	HLH	HLC	HLE	HLM
	**% Yield**	1.65	1.59	0.37	1.46

* The three letter codes represent the 'plant, plant part, and solvent' respectively e.g. MLH represents '*Margaritaria discoidea*, leaves, hexane' extract. The extraction solvent and plant part letter codes are shown in parentheses. The yields of crude extract range from 0.18% in *Margaritaria discoidea *stem bark hexane extract (MBH) to 2.12% in *Margaritaria discoidea *leaves methanol extract (MLM).

### Preparation of plant extracts

The leaves, stem bark and roots of *M. discoidea*, and the leaves of *H. africanum *were dried in an oven (40°C), and ground to fine powder. Each powder was weighed and macerated for 48 hours, three times per solvent and sequentially in hexane, methylene chloride, ethylacetate and methanol. The mixture was filtered and the filtrate concentrated under reduced pressure by rotary evaporation (BUCHI Rotavapor R-200, Switzerland) at appropriate temperatures. Residual solvent was removed by drying in air at room temperature (23 - 25°C) and the extract weighed and stored at -20°C until used.

### Isolation of *O. ochengi *adult worms

The isolation of *O. ochengi *adult worms was done by the method of Cho-Ngwa *et al*. [11]. Briefly, fresh pieces of umbilical cattle skin with palpable nodules bought from local slaughterhouses were washed, drained and sterilized with 70% ethanol. *O. ochengi *adult worms were carefully scraped out of the nodules as single masses and temporarily submerged in complete culture medium, CCM [RPMI-1640 (SIGMA, USA) supplemented with 25 mM HEPES, 2 g/L sodium bicarbonate, 2 mM L-glutamine, 5% new born calf serum (SIGMA, USA), 150 units/mL penicillin, 150 μg/mL streptomycin and 0.5 μg/mL amphotericin B (SIGMA, USA), pH 7.4)] for 1 hour prior to the assay in order to eliminate dead microfilariae. Damaged worms and worms from putrefied nodules were discarded. The viability of worms retained for the assay was ascertained by visual and microscopic examination of adult worm and microfilarial motility using an inverted microscope (Euromex, Holland).

### Isolation of *O. ochengi *microfilariae

The cattle skin was obtained as previously described [11]. About 5 skin snips were obtained from different locations of the skin and incubated separately in small amounts of CCM for 30 minutes. Emerged microfilariae (mfs) were qualified and quantified for *O. ochengi *microfilariae with the aid of an inverted microscope. A selected piece of skin was carefully shaved with a razor blade, and then rinsed with distilled water. It was dabbed with a clean tea cloth to eliminate excess moisture, and covered entirely with 70% ethanol. The latter was allowed to evaporate completely in a horizontal flow sterile hood. The ethanol treatment was repeated once. The duration from the slaughtering of a cow to the harvesting of parasites from the skin was always less than 2 hours to avoid bacterial contamination. The sterilized skin was tautly attached onto an autoclaved, cylindrical piece of wood using autoclaved thumb nails and close (about 1 mm apart) criss-cross cuts were made into the epidermis and dermis. The assembly was incubated in the culture medium for 4-6 hours. The emerged and highly motile *O. ochengi *microfilariae were concentrated by centrifugation at 400 × *g *for 10 minutes and quantified.

### Preparation of mammalian cells

Monkey kidney epithelial cells (LLC-MK_2_) (ATCC, USA) were cultured at 37°C in humidified air with 5% CO_2 _in a HeraCell-150 incubator (Thermo Electron, Germany) until the cell layer became fully confluent. The cells were rinsed with a solution of 0.125% trypsin and 0.5 mM EDTA in medium 199 (Sigma, USA) and kept in the same mixture for less than 1 hour for them to be dislodged. The cell suspension was centrifuged at 560 × g for 10 minutes, the supernatant discarded and the pellet re-suspended to 2 × 10^5 ^cells/mL in CCM. The cell suspension was dispensed into 96-well microtitre plates (200 μL/well) and kept in the incubator for 3 - 5 days for cells to grow and become fully confluent. These cells served as feeder layer for the mfs assays and were also used for cytotoxicity studies.

### Preparation of stock solutions of plant extracts

Twenty-five milligrams (25 mg) of each crude extract was weighed and dissolved in microtubes containing 1 mL of 100% hybrimax™ dimethyl sulfoxide (DMSO) (SIGMA, USA) to obtain stock solutions of 25 mg/mL. Complete dissolution was achieved by vortexing. The solutions were then filtered through DMSO-safe 0.20 μm sterile filters (Corning, USA) and stored at 4°C for one week before they were used in the assays.

### Anti-filarial screening of plant extracts

#### Primary screen on adult worms

This was done to eliminate inactive extracts. Adult worm assays were conducted in 24-well plates (NUNC, USA) at 37°C in humidified air containing 5% CO_2 _for 5 days (120 hours) without change of medium. Nodular masses (each generally containing a few males and a female worm) were first put in the wells (with 2 mL CCM) without extract overnight to confirm their viability. The crude extracts were then added in quadruplicate wells at a single final concentration of 500 μg/mL by substituting 1 mL of the medium in the well with 1 mL of CCM containing 1000 μg/mL of extract. Six nodular masses each, were used in the negative (2% DMSO only) and in the positive (amocarzine-CGP 6140, 10 μg/mL) control wells in which each well received only one nodular mass. Adult worm viability was assessed by the MTT/formazan assay in which each nodular mass was placed under sterile conditions in a well of a 96-well microtitre plate containing 200 μL/well of 0.5 mg/mL MTT (Sigma, USA) in phosphate buffered saline (PBS), and then incubated under the culture conditions. Adult worm viability was taken as least % inhibition of formazan formation relative to negative control at 120 hours post addition of drug. An extract was considered active if there was a 90% or greater inhibition of formazan formation compared to the negative controls; or moderately active if the inhibition was 50 - 89%. It was considered inactive if the inhibition was less than 50%.

#### Primary screen on microfilariae

The extracts were also tested at a single concentration of 500 μg/mL, in duplicate wells. The mfs assay was conducted in 96-well microtitre plates (15 mfs in 200 μL CCM per well) at 37°C in humidified air containing 5% CO_2 _for 5 days without any change of medium. Fully confluent monkey kidney epithelial cells, serving as feeder layer, were co-cultured with the mfs. The medium used in preparing the feeder cell layer was removed by a swift decantation before fresh CCM containing drug (100 μL) and worms (100 μL) were immediately added. Amocarzine (10 μg/mL) and 2% DMSO served as the positive and negative controls respectively. Mfs motility scores (viability) were done on a scale of 0 (immotile), through 0.25 (only tail or head shaking occasionally), through 0.5 (whole body motile, but sluggishly or with difficulties), to 1 (almost vigorous to vigorous motility). Scores were made every 24 h, terminating at 120 h using an inverted microscope. Any culture with microbial contamination was not considered. Mfs viability was taken as the mean % reduction at 120 h (day 5) after addition of drug. An extract was considered active if there was a 100% reduction in mfs motility compared to the control; or moderately active for a motility reduction of 50 - 99%; and inactive if the reduction was less than 50%.

#### Secondary screen on microfilariae

This was done to confirm the activity of extracts that showed filaricidal activity in the primary screen, and to determine their IC_50_, IC_100 _and selectivity index (SI) values. Since no extract showed macrofilaricidal activity in the primary screens, the secondary screen was limited to the mfs. The extracts were retested as described under primary screens at serial dilutions from 500 to 3.91 μg/mL. The graphical analysis was done using Microsoft Excel 2007 (Microsoft Corporation, USA).

### Toxicity studies

#### Cytotoxicity studies

This was done as part of the mfs assay on the active extracts through observations on the monkey kidney epithelial cells on day 5. An examination of the shapes of the monkey kidney cells was also done. Dead or deformed cells were usually detached from the bottom of the vessel and were rounded in shape. The IC_50 _values for these mammalian cells were determined graphically using data from microscopy. The selectivity index (SI) values were calculated using the ratio:

SI = IC_50 _mammalian cell/IC_50 _parasite (mfs)

#### Acute toxicity test

This test was conducted in accordance with the European Economic Community (EEC) guidelines regarding the protection of animals used for experimental and other scientific purposes [12]. The two extracts with SI values greater than 2 were tested for acute toxicity in Balb/c mice (both males and females of approximately 20 g body weight each) at a dose of 5 × IC_50_. The required amount of each extract was dissolved in 100% DMSO and then diluted in RPMI-1640 medium to a final DMSO concentration of 2%. Then 4.5 mg of MRH in 0.9 mL of diluent was injected into the peritoneal cavity of each 20 g mouse; while 9.5 mg of HLH in 0.95 mL of diluent was injected per mouse in the same way. The control group received the same amount of diluent only, and was kept under the same conditions as the test mice. Six mice were used per treatment or control group. The animals were observed for physical activity, food and water intake, loss of fur, general behaviour and mortality for 14 days.

### Phytochemical analysis

The presence of the major phytochemical derivatives in the extracts with SI values greater than 2 were investigated using standard methods. Briefly, sterols and terpenes were tested by thin-layer chromatography on aluminium plates coated with silica gel. One half gram (0.5 g) of each extract was tested. The mobile phase was a hexane/ethylacetate (8:2) mixture. The spots were developed by spraying with a conc. H_2_SO_4_/H_2_O (1:1) mixture followed by heating until pink and yellow spots appeared on the positive control lanes. For saponins, about 0.5 g of each extract was put in a test tube and water was added and shaken to observe foaming, characteristic of saponins. Other tests carried out were for anthraquinones (Borntrager's test, [13]); terpenoids (Salkowski test), flavonoids (Shinoda's test), and alkaloids [14].

## Results

### Activity of *M. discoidea *and *H. africanum *extracts in primary screens

Of the 16 extracts prepared from the various plant parts and tested at 500 μg/mL, 2 from *M. discoidea *and 2 from *H. africanum *were microfilaricidal (Table 2), while none were active against the adult worms. At 10 μg/mL, amocarzine produced a 100% inhibition of mfs motility at 24 hours of incubation. The hexane extract of *H. africanum *leaves (HLH), the hexane extract of *M. discoidea *roots (MRH), the methylene chloride extract of *H. africanum *leaves (HLC) and the methylene chloride extract of *M. discoidea *leaves (MLC) all produced 100% inhibition at 500 μg/mL. These 4 extracts were then retested against mfs in the secondary screens.

**Table 2:** Effect of extracts on the viability of microfilariae at 120 hours in secondary screens

Concentration of extract (μg/mL)	% Inhibition of microfilarial motility				
	
	MRH	MLC	HLH	HLC	Negative control*
**500**	100	100	100	100	0
**250**	100	100	100	100	0
**125**	100	100	100	100	0
**62.5**	100	50	0	100	0
**31.25**	0	0	0	50	0
**15.63**	0	0	0	0	0
**7.81**	0	0	0	0	0
**3.91**	0	0	0	0	0
**0**	0	0	0	0	0

All four extracts codes are defined in Table 1. *The negative control wells contained worms in culture medium with 2% DMSO only.

### Activity of *M. discoidea *and *H. africanum *extracts in secondary screens, and toxicity studies

**Figure 1:**
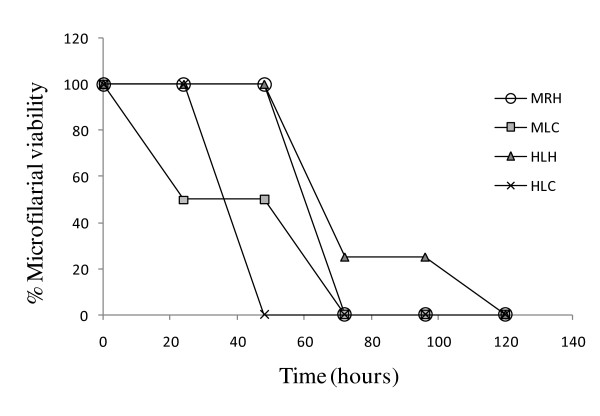
**Effect of active extracts on the viability of *O. ochengi *microfilariae over time**. All extracts were tested at their IC_100_. MRH = hexane extract of *M. discoidea *roots; MLC = methylene chloride extract of *M. discoidea *leaves; HLH = hexane extract of *H. africanum *leaves; and HLC = methylene chloride extract *H. africanum *leaves

Table 3 summarises the results obtained. HLC and MRH demonstrated the highest activity with 100% inhibition of mfs motility at 62.5 μg/mL and HLC showed the least IC_50 _of 31.25 μg/mL. On the contrary, HLH recorded the highest IC_50 _of 95 μg/mL. Generally, the percentage inhibition of mfs motility increased with concentration of extract and with time of incubation in drug (Figure 1). Hence, all the extracts exhibited a dose and time dependent inhibition of mfs motility (Table 2; Figure 1). Furthermore, all the extracts (except MLC), were more toxic to mfs than to mammalian cells as reflected in their IC_50 _values (Table 3). HLH recorded the highest SI of 2.63 while MLC recorded the lowest SI of 0.5 (Table 3). Thus, MLC was more toxic to cells than to parasites, and its anti-parasitic activity could be due to this general toxicity. The two active extracts with SI values greater than 2 showed no form of acute toxicity at 5 × IC_50 _in Balb/c mice (Table 3).

**Table 3:** IC_50_, IC_100 _and SI of active extracts on the microfilariae of *O. ochengi*

Extract	Mammalian cells	Microfilariae			Acute toxicity in mice at 5 × IC_50 _on mfs
		
	IC_50 _(μg/mL)	IC_50 _(μg/mL)	IC_100 _(μg/mL)	SI	
**MRH**	93.75	45	62.5	2.08	Non-toxic
**MLC**	31.25	62.5	125	0.5	Not determined
**HLH**	250	95	125	2.63	Non-toxic
**HLC**	46.88	31.25	62.5	1.5	Not determined

SI - Selectivity index. The codes of the extracts are: MRH = hexane extract of *M. discoidea *roots; MLC = methylene chloride extract of *M. discoidea *leaves; HLH = hexane extract of *H. africanum *leaves; HLC = methylene chloride extract *H. africanum *leaves.

### Phytochemical analysis

Anthraquinones, sterols and terpenoids were shown to be present in the two extracts having SI values greater than 2 (Table 4).

**Table 4:** Phytochemical screening of active extracts with selectivity index values greater than two

Class of Compound	*H. africanum Extract *HLH	*M. discoidea Extract *MRH
Saponin	-	-
Anthraquinones	-	+
Sterols	+	+
Terpenoids	+	+
Alkaloids	-	-
Flavonoids	-	-

+: present; -: absent. The codes of the extracts are: MRH = hexane extract of *M. discoidea *roots; HLH = hexane extract of *H. africanum *leaves

## Discussion

The aim of this study was to screen crude extracts of *M. discoidea *and *H. africanum *plants that are popularly used in the traditional treatment of onchocerciasis in North West Cameroon for their activities against *O*. *ochengi *and as potential sources of novel *O. volvulus *filaricidal lead compounds. The lack of a suitable macrofilaricide for the treatment of human onchocerciasis and the emergence of ivermectin resistant nematodes of veterinary importance underscores the urgency for novel drugs/phytomedicines or lead compounds. Previous assays for drug screens in onchocerciasis have been based on the use of *O. lienalis *or *O. gutturosa *worms [15] which are not as closely related to *O. volvulus *as is *O. ochengi*, or on use of *O. volvulus *itself which is expensive and difficult to obtain from infected humans through surgery. The *O. ochengi *model is now considered the most suitable for screening for anti-*O. volvulus *drugs because of the similarities between both parasites which share the same *Simulium *vector, and are equally susceptible to ivermectin and suramin [16], in addition to the availability and relatively low cost of obtaining *O. ochengi *[17].

Sixteen (16) crude extracts of different polarities from the 2 plant species were tested. Four (4) of the extracts showed activity on microfilariae only (Table 3), while none showed activity on adult worms in the primary screen. The remaining 12 extracts were inactive on adult worms and mfs. All 4 microfilaricidal extracts were non-polar. It is thus likely that the active compounds in *M. discoidea *and *H. africanum *are predominantly non-polar. This corroborates previous studies which showed that non-polar compounds such as essential oils are nematocidal [18]. The root extracts of *M. discoidea *exhibited a higher activity against mfs than the leaves as seen from the IC_50 _and IC_100 _values of 45 μg/mL and 62.5 μg/mL versus 62.5 μg/mL and 125 μg/mL for the roots and leaves respectively. The roots extract also had a higher SI value compared to the leaves. On the other hand, only the leaves of *H. africanum *were used in this study as is the practice in traditional medicine of the people concerned. The methylene chloride extract of *H. africanum *(HLC) recorded the overall best activity with the least IC_50 _and IC_100_. Interestingly, this extract recorded an SI of 1.5 (Table 3). An SI value of greater than 1 for a crude extract increases the likelihood that its toxic and filaricidal components are different. Thus, elimination of these toxic components may yield more suitable filaricidal drug leads or phytomedicines. Also, the higher the SI value, the greater the probability of isolating safe leads from an extract. Where the anti-parasitic IC_50 _is low, and the SI value is high, the formulation of a phytomedicine for local use is encouraged.

The acute toxicity studies in mice revealed that the two active extracts, MRH and HLH with SI values greater than 2 (Table 3) are non-toxic at 5 × IC_50 _despite their relatively low SI values. This may imply a detoxification mechanism in the liver of mice *in vivo*. One other study [19] also did not find any acute toxicity and no adverse change in behavior in mice following oral administration of an aqueous extract (3200 mg/kg body weight) of *M. discoidea*. This finding in mice lends credence to the ethnopharmacologically observed lack of toxicity or adverse effects in humans.

In addition to the compound groups detected in the promising extracts (MRH and HLH) (Table 4), other workers have reported the presence of alkaloids in these plants [10]. Alkaloids may be found in other parts of the plant (not tested by us) as indicated in the literature. Some of these compounds may be responsible for the filaricidal activity of the plant extracts. However the purification and further antifilarial screening of the pure compounds from the extracts will be required to determine their full potential.

Overall, the effect of the active extracts on mfs increased with incubation time (Figure 1), and there was a general decrease in % viability of mfs with increasing extract concentration, suggesting a dose dependent action. However, the observation that the extracts MRH, MLC, HLH and HLC were only microfilaricidal and not macrofilaricidal could probably be due to lack of the target for the active components in the adult worms.

## Conclusions

The non-polar extracts of *M. discoidea *and *H. africanum *roots and/or leaves are potential sources of new microfilaricidal compounds for the treatment of onchocerciasis. The results obtained also support their use in traditional medicine and in the preparation of phytomedicines against *Onchocerca *microfilariae.

## Competing interests

The authors declare that they have no competing interests.

## Authors' contributions

FCN did the conception, design, and supervision of the experiments. He carried out many of the culture experiments, as well as analysed and interpreted the data. MA collected the plants, prepared the extracts, performed some of the experiments and contributed in drafting the manuscript. MN and KDN contributed to the study design, plant collection, identification and extraction, analysis and interpretation of the data and drafting of the manuscript. All authors read the manuscript, contributed in correcting it, and approved its final version.

## Pre-publication history

The pre-publication history for this paper can be accessed here:

http://www.biomedcentral.com/1472-6882/10/62/prepub
